# Image Enhancement for Computational Integral Imaging Reconstruction via Four-Dimensional Image Structure

**DOI:** 10.3390/s20174795

**Published:** 2020-08-25

**Authors:** Joungeun Bae, Hoon Yoo

**Affiliations:** 1 Department of Computer Science, Sangmyung University, 20 Hongjimoon-2gil, Seoul 030031, Korea; wjddms5810@naver.com; 2 Department of Electronics Engineering, Sangmyung University, 20 Hongjimoon-2gil, Seoul 030031, Korea

**Keywords:** integral imaging enhancement, 3-D computational reconstruction, image enhancement

## Abstract

This paper describes the image enhancement of a computational integral imaging reconstruction method via reconstructing a four-dimensional (4-D) image structure. A computational reconstruction method for high-resolution three-dimensional (3-D) images is highly required in 3-D applications such as 3-D visualization and 3-D object recognition. To improve the visual quality of reconstructed images, we introduce an adjustable parameter to produce a group of 3-D images from a single elemental image array. The adjustable parameter controls overlapping in back projection with a transformation of cropping and translating elemental images. It turns out that the new parameter is an independent parameter from the reconstruction position to reconstruct a 4-D image structure with four axes of *x*, *y*, *z*, and *k*. The 4-D image structure of the proposed method provides more visual information than existing methods. Computer simulations and optical experiments are carried out to show the feasibility of the proposed method. The results indicate that our method enhances the image quality of 3-D images by providing a 4-D image structure with the adjustable parameter.

## 1. Introduction

**Figure 1:**
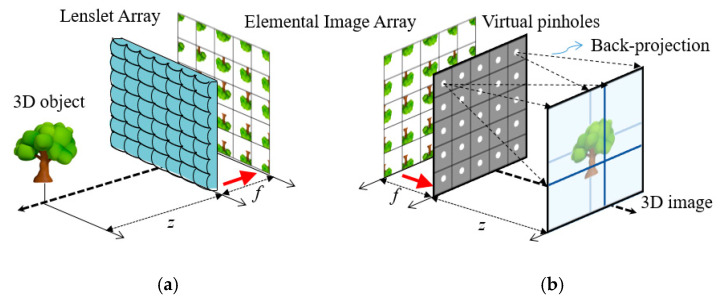
Computational integral imaging system (**a**) pickup (**b**) computational reconstruction.

Integral imaging is a promising technique for three-dimensional (3-D) imaging and visualization, which was proposed by Lippmann in 1908 [1]. Compared with 3-D imaging techniques based on stereoscopy or auto-stereoscopy, integral imaging provides advantages such as full parallax with white light and continuous viewpoints without wearing eyeglasses. Thus, integral imaging has received considerable attention in various applications such as 3-D visualization and 3-D object recognition [2,3,4,5,6,7,8]. In general, an integral imaging system consists of a pickup and a reconstruction process, as illustrated in Figure 1. The pickup process records light rays coming from a 3-D object through a lens array on a camera or a camera array. An array of different perspectives of a 3-D object is called an elemental image array (EIA). On the other hand, the reconstruction process reconstructs a 3-D object from an elemental image array optically or digitally. Optical reconstruction is displaying 3-D objects from their elemental image array through a lens array. Computational reconstruction generates a volume of 3-D objects digitally. This reconstruction method is called the computational integral imaging reconstruction (CIIR). A CIIR method produces a volume of 3-D objects without optical limitations such as lens aberrations and the barrel distortion. The volume from a CIIR method is practical to recognize 3-D objects and to estimate their depths.

Normally, CIIR methods are based on the back projection [9,10,11,12,13,14,15,16,17,18,19,20,21,22,23,24,25,26,27,28,29]. The principle of the back- projection is that 2-D elemental images are projected over 3-D space then each projected image is overlapped each other at a reconstruction image plane [9,10,11]. Due to its simple model for the ray optics, the back projection-based CIIR methods have been actively studied to enhance 3-D images [12,13,14,15,16,17,18,19,20,21,22,23,24,25,26,27,28,29]. Those methods can be classified into pixel-mapping-based projection, windowing-based projection, convolution-based projection. Pixel mapping methods project each pixel of an elemental image array passing through the pinhole array into 3-D space [12,13,14,15,16]. Those methods reduced computational costs and improved the visual quality of the reconstructed images. The windowing methods project weighted elemental images into 3-D space, where windowing functions are defined from a signal model of CIIR. The signal model improved the visual quality of the reconstructed images by removing blurring and lens array artifacts [17,18]. Recently, CIIR methods based on convolution and the delta function have been reported to obtain depth information. The methods can provide enhancement of reconstructed images and control of the depth resolution [19,20,21,22,23,24,25]. Additionally, CIIR methods using tilted elemental image array were proposed to improve image quality [26,27]. A depth-controlled computational reconstruction with sub-images or continuously non-uniform shifting pixels was proposed to obtain improved depth resolution and image quality [28,29].

In this paper, we propose a novel CIIR method that generates a four-dimensional (4-D) image structure to enhance the visual quality of reconstructed images. Our method reconstructs a group of 3-D images from an elemental image array by introducing a new parameter *k*. The new parameter is defined as an overlapping number in back projection. Formulas are proposed to use the parameter to transform an elemental image array with cropping and translating. Here, the cropping ratio for each elemental image is calculated from a given adjustable parameter *k*. Our method is capable of generating a group of the reconstructed images at the same depth through varying the parameter; thus, it reconstructs a group of 3-D images from a single elemental image array. Then, we define the 3-D images by varying the parameter *k* as a 4-D image structure. The 4-D structure of the proposed method can provide more visual information as well as improve the visual quality of the 3-D images. To evaluate the proposed method, computer simulation and an optical experiment are conducted. The experimental results indicate that the proposed method enhances the visual quality of the reconstructed images and provides additional visual information with the 4-D image structure.

## 2. Conventional Computational Integral Imaging Reconstruction

**Figure 2:**
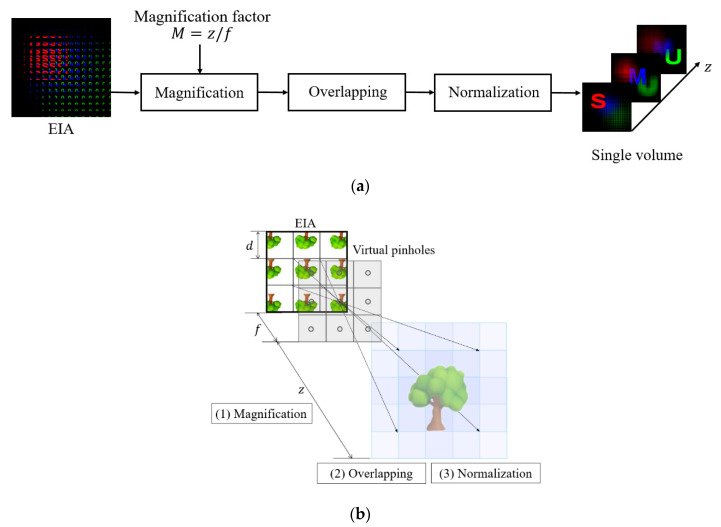
(**a**) Process of reconstructing a volume in the conventional CIIR method. (**b**) The conventional CIIR method for an elemental image array.

Computational integral imaging reconstruction (CIIR) is a method of converting an EIA into a 3-D volume. The conventional CIIR method consists of magnification, overlapping, and normalization, as depicted in Figure 2. In magnification, each elemental image is back projected through its virtual pinhole on 3-D space where it is magnified with a magnification factor of *M*. The magnification factor is determined by *M* = *z*/*f*. Here, *z* is the distance away from virtual pinholes and *f* is the distance between an elemental image array and its virtual pinhole. The distance *f* is also considered to be the focal length of the virtual pinhole. In overlapping, the magnified elemental images are overlapped at a reconstructed image plane. Let an overlapping number be the number of incoming rays at the reconstructed image plane. Each pixel of the reconstructed image plane has a maximum overlapping number of *M*^2^ when the magnification factor is *M*. It also has an uneven overlapping number. Thus, normalization is required to eliminate the uneven overlapping in the plane [17].

The maximum overlapping number in a reconstruction plane increases in proportion to a depth of *z* since magnifying elemental images in proportion to *z* causes severe overlapping. Overlapping of elemental images focuses the 3-D objects on a reconstruction plane, which we called in-focus overlapping. However, the focused 3-D object can be blurred due to the overlapping if the elemental image array obtained from a lens array suffers from noises, lens aberrations, and lens distortion. Reducing the blurring of in-focus overlapping is required to improve the visual quality in 3-D imaging.

## 3. Computational Integral Imaging Reconstruction by Multiple Volume Reconstruction

**Figure 3:**
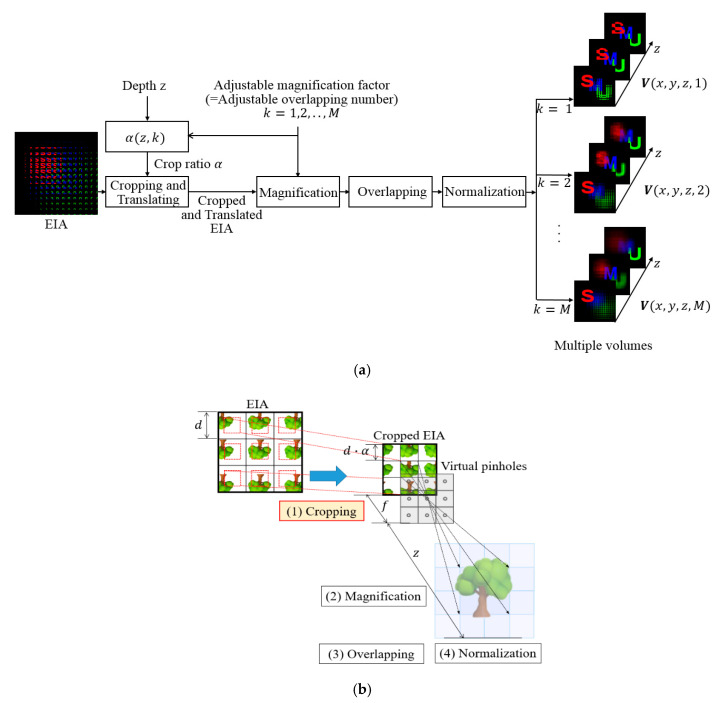
(**a**) Process of reconstructing multiple volumes in the proposed CIIR method (**b**) The proposed CIIR method for an elemental image array.

The proposed CIIR method consists of four processes: cropping, magnification, overlapping, and normalization, as illustrated in Figure 3. The main difference between the conventional method and the proposed method is the introduction of a new adjustable magnification factor that is denoted by *k*. The adjustable magnification factor is equal to controlling the crop ratio of the elemental images. The conventional CIIR method reconstructs a single volume where the magnification factor depends on the depth of each reconstruction plane. The overlapping number of elemental images is also equal to the magnification factor. Ideal overlapping produces a focused object at a specific location. However, overlapping can cause a blurring of the focused object due to optical pickup devices. We propose a free parameter of the magnification factor to control the overlapping number. The free magnification factor is considered to the additional axis of *k*, which reconstructs a 4-D image structure that is denoted by ***V***(*x*, *y*, *z*, *k*). Thus, the proposed method enables the blurring of a focused object to be reduced by controlling the overlapping number. Besides, our method can provide enhanced visual information from our 4-D structure.

**Figure 4:**
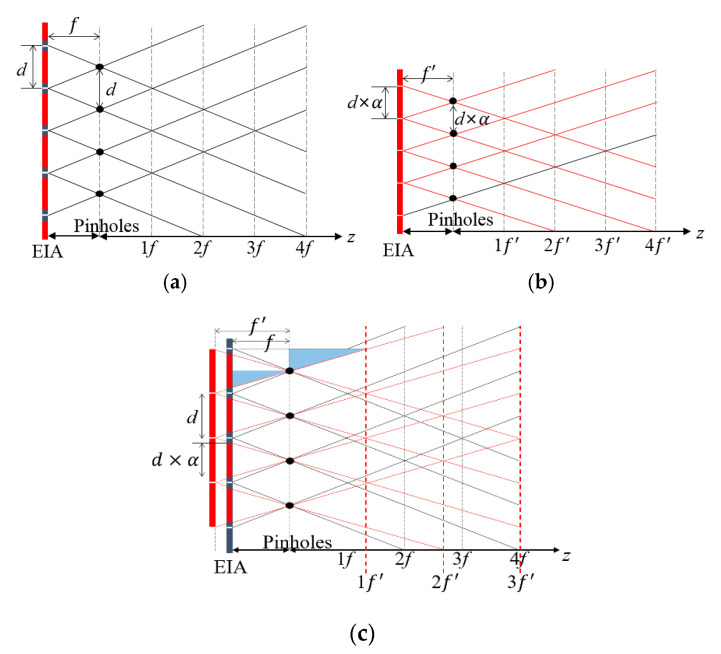
Model of the proposed method of reconstructing cropped elemental images (**a**) reconstruction model of the original EIA (**b**) reconstruction model of the cropped EIA (**c**) reconstruction model of the original EIA and cropped EIA.

Figure 4 illustrates the principle of adjusting the magnification factor by cropping elemental images in our method. The model of conventional computational reconstruction is depicted in Figure 4a, where each elemental image size is *d* and the focal length is *f*. When these elemental images are cropped, a crop ratio is denoted by *α* where 0 < *α* ≤ 1. The new model of a computational reconstruction with cropped and translated elemental images is shown in Figure 4b, where each elemental image size and the pitch of pinholes are converted to *d* × *α*. Thus, the focal length of the new model can be changed relatively. Let us denote a new focal length by *f*′. It is seen that an elemental image array is transformed into a new elemental image array. The transformation between two elemental image arrays consists of a cropping process and a translation process. Let each reconstructed image from each model at the physically same depth be identical. Then, the pitch between pinholes from the new model matches the pitch from the conventional model, as shown in Figure 4c. The triangular similarity between two blue triangles in Figure 4c yields a new focal length

(1) $$f^{\prime }=\frac{f}{\alpha }$$

It says that cropping elemental images enlarges the focal length. Then, the depth is represented by *z* = *kf*′ with a new focal length and a new magnification factor *k*. When the depth of *z* = *Mf* is the same as that of *z* = *kf*′, the crop ratio is evaluated using (1) as

(2) $$\alpha \left(z,k\right)=\frac{f}{f^{\prime }}=\frac{k}{M}=\frac{kf}{z}$$

where 1≤ *k* ≤ *floor*(*M*) and an integer. This equation indicates that the crop ratio is adjustable to control the magnification factor of *k*, where the factor *k* and the depth *z* are independent and the focal length *f* is a fixed value. Note that the magnification factor of *M* depends on the depths in the conventional method. On the other hand, the magnification factor of *k* is a free parameter in our method. The free parameter of *k* is also a visual parameter of the reconstructed image since it controls the overlapping numbers. The adjustable magnification factor is considered to the additional axis of *k*, which reconstructs a 4-D image structure. Let us define a 4-D image structure as ***V***(*x*, *y*, *z*, *k*). Additionally, let us define a 3-D-reconstructed image as *V_k_*(*x*, *y*, *z*) or *V_k_*, where all images have the same overlapping number of *k*.

**Figure 5:**
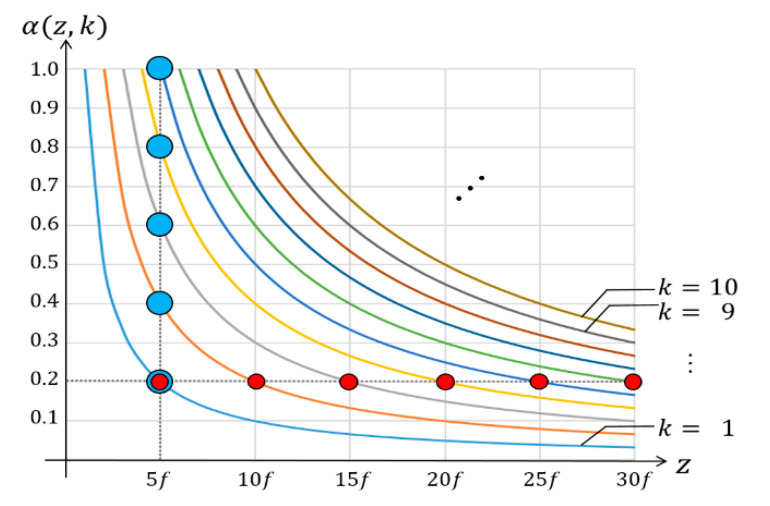
Graph of crop ratio *α* versus magnification factor *k* at the depth *z* in the proposed CIIR.

Figure 5 shows the graph of the crop ratio with respect to the magnification factor and the depth, according to (2). For example, *α* versus *k* for a fixed depth of *z* = 5*f* are highlighted by blue circles in the graph. The crop ratio of *α* increases by a step of 0.2 when the magnification factor of *k* increases by a step of 1. As another example, *k* versus *z* for a fixed crop ratio of 0.2 is shown as red circles in the graph. The magnification factor of *k* increases by a step of 1 when the depth of *z* increases by a step of 5*f*. The graph indicates that our method obtains the crop ratio with the specific magnification factor at the specific depth.

## 4. Simulations and Discussions

**Figure 6:**
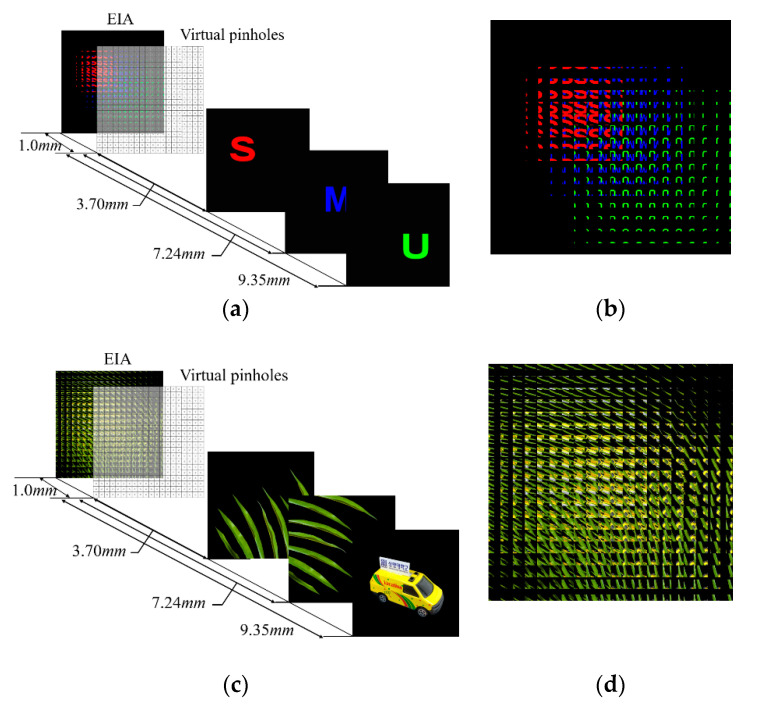
(**a**) The images of ‘S’, ‘M’, and ‘U’ used for simulation, (**b**) generated EIA of ‘S’, ‘M’, and ‘U’, (**c**) the images of ‘leaves’ and ‘car’ used for simulation, (**d**) generated EIA of ‘leaves’ and ‘car’.

Computer simulations are conducted to objectively evaluate the proposed computational reconstruction method with the adjustable magnification factor. The proposed method is compared with the standard method [11] in terms of subjective and objective measures. Test objects of ‘S’, ‘M’, ‘U’, ‘leaves’, and ‘car’ and their elemental image arrays (EIAs) are shown in Figure 6. The number of elemental images is 16 × 16 in the EIAs, and each elemental image consists of 100 × 100 pixels. The gap between the virtual pinhole array and the elemental image array is set to be *f* = 1.0 mm. The locations of the test objects away from the virtual pinhole array are 3.70, 7.24, and 9.35 mm.

**Figure 7:**
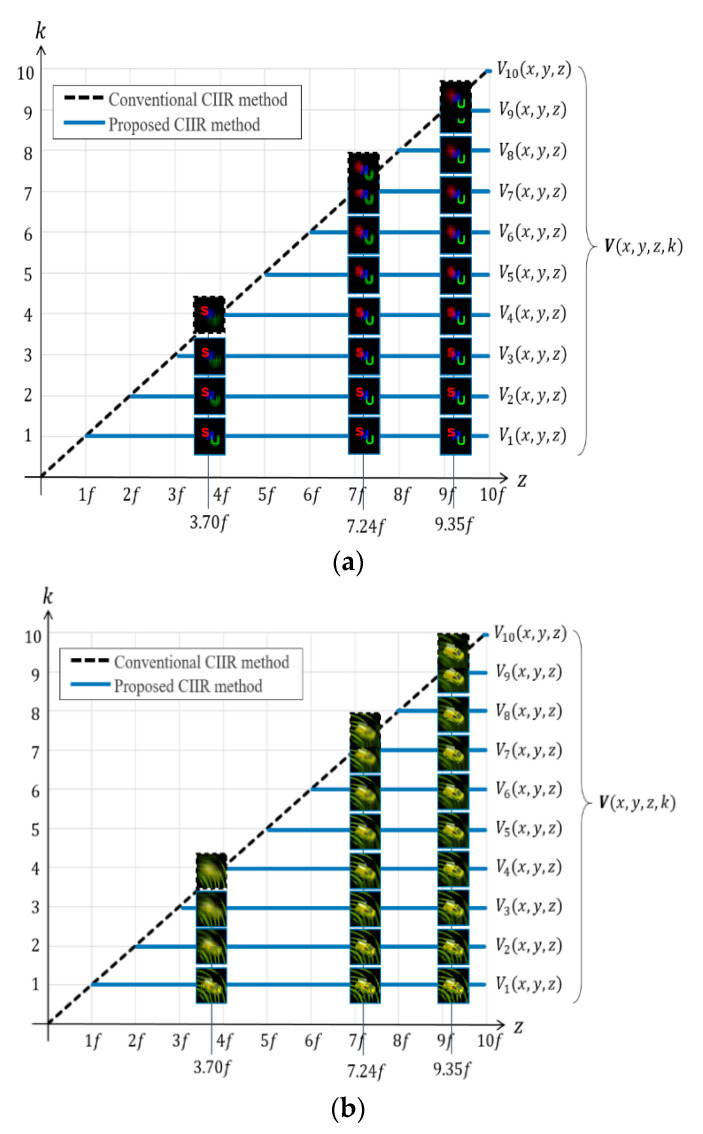
Reconstruction locations adjusting *k* and *z*. A 2-D array of reconstructed images from the test object (**a**) ‘S’, ‘M’, and ‘U’, (**b**) ‘leaves’ and ‘car’.

Figure 7 shows the reconstruction locations adjusting a magnification factor *k* and a depth *z*, especially the depths are *z* = 3.70*f*, 7.24*f*, and 9.35*f*. The proposed method reconstructs a group of images for each depth by varying the adjustable parameter *k*; thus, two-dimensional arrays of two-dimensional images are shown in Figure 7a,b. On the contrary, the conventional method [11] reconstructs an image for each depth, as shown on the diagonally dashed lines in Figure 7. Therefore, multiple volumes are obtained from the proposed computational by adding a new axis of *k* to the axis of *x*, *y*, and *z*, whereas a single volume is from the conventional method.

**Figure 8:**
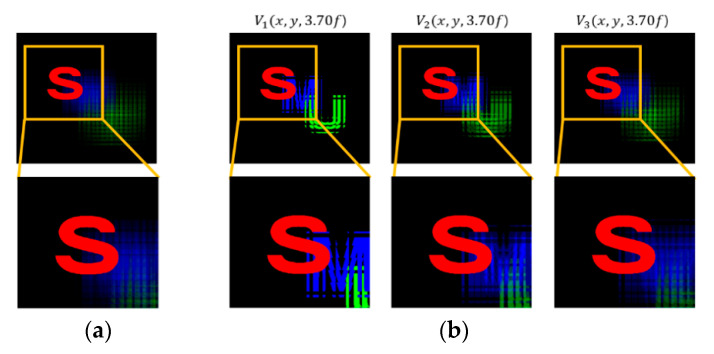
Reconstructed images for the object ‘S’ at a depth of *z* = 3.70*f* with (**a**) conventional CIIR [11], (**b**) proposed CIIR.

**Figure 9:**
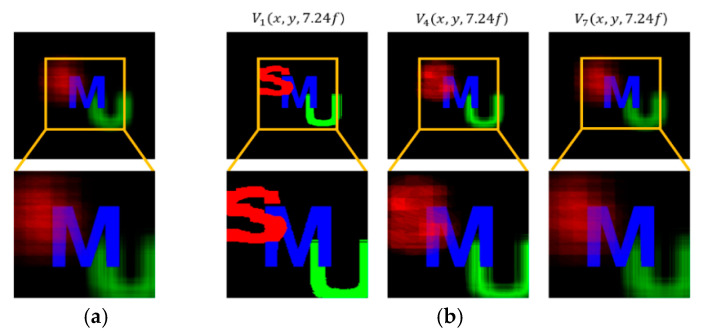
Reconstructed images for the object ‘M’ at a depth of *z* = 7.24*f* with (**a**) conventional CIIR [11], (**b**) proposed CIIR.

**Figure 10:**
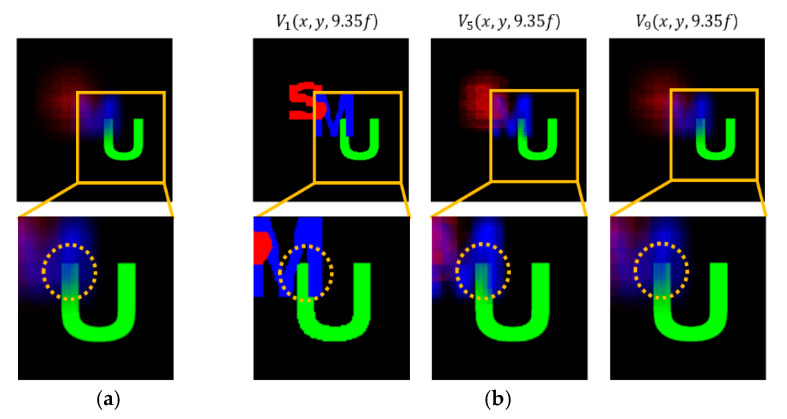
Reconstructed images for the object ‘U’ at a depth of *z* = 9.35*f* with (**a**) conventional CIIR [11], (**b**) proposed CIIR.

**Figure 11:**
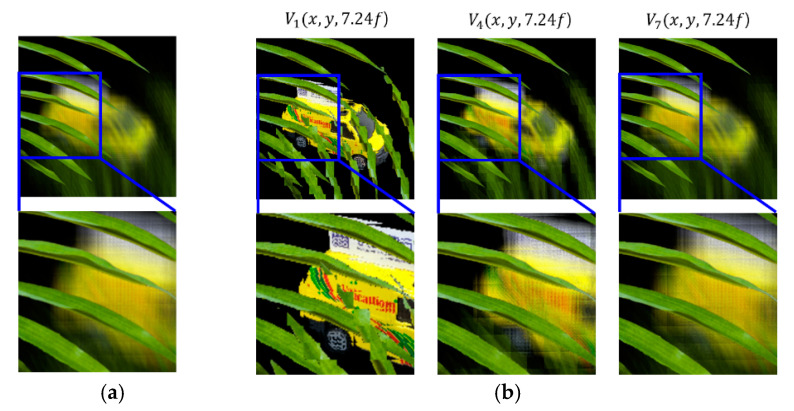
Reconstructed images for the object ‘leaves’ at a depth of *z* = 7.24*f* with (**a**) conventional CIIR [11], (**b**) proposed CIIR.

**Figure 12:**
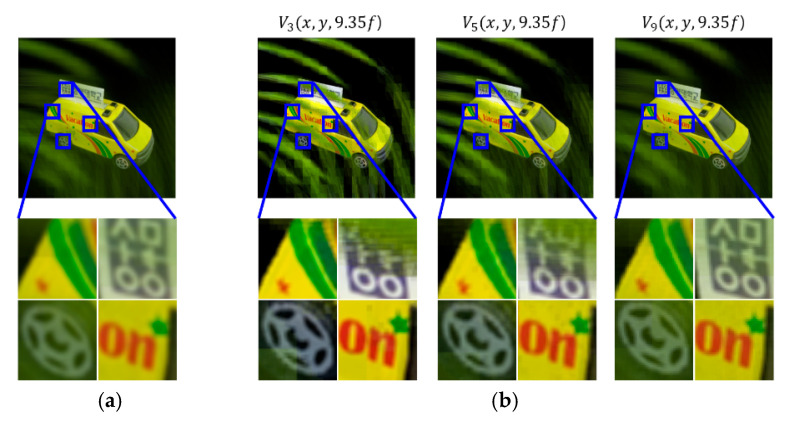
Reconstructed images for the object ‘car’ at a depth of *z* = 9.35*f* with (**a**) conventional CIIR [11], (**b**) proposed CIIR.

A set of reconstructed images from the conventional method and the proposed method is shown in Figure 8, Figure 9, Figure 10, Figure 11 and Figure 12, where reconstruction depths are *z* = 3.70*f*, 7.24*f*, and 9.35*f*. The conventional method provides a reconstructed image at each depth, as shown in Figure 8, Figure 9, Figure 10, Figure 11 and Figure 12a. The proposed method provides three different images at each depth by varying *k*, as shown in Figure 8, Figure 9, Figure 10, Figure 11 and Figure 12b. Here, the reconstructed images for each depth from the proposed method have different contents for the same object. It is seen that varying the adjustable parameter *k* is controlling the depth of focus in a reconstructed image. In the previous method, a reconstructed image at a given depth shows that the object area at the same depth is focused and elsewhere is defocused. However, the proposed method enables us to control this focusing property with a free variable. Thus, this property of the proposed method provides more information with a given EIA than the previous method.

It is seen that the reconstruction characteristic according to *k* is shown in Figure 8, Figure 9 and Figure 10b. Here, increasing *k*, which means the overlapped areas increase, offers to reduce the depth of focus. On the contrary, decreasing *k*, reducing the overlapped areas, enables wider areas to be focused. For example, the image of *V*_1_(*x*, *y*, 9.35*f*) with a factor of *k* = 1 has no blurred objects because there is no overlapping, as highlighted with yellow circles in Figure 10b. The objects ‘S’ and ‘M’ reconstructed at mismatched positions seem to be fragmented rather than blurred. The object ‘U’ located at the right distance is reconstructed without fragmenting. Additionally, the occluding effect from other objects is minimized due to the fact there is no overlapping. For another example, as shown in Figure 10, the image of *V*_9_(*x*, *y*, 9.35*f*) with a factor of *k* = 9 is overall recognized though it is partially blurred and loses the information that is seen in the image with a factor of *k* = 1. Similarly, as shown in Figure 12b, some areas in the reconstructed image *V*_3_(*x*, *y*, 9.35*f*) with a factor of *k* = 3 for the object ‘car’ have high-quality scenes partially, whereas the image *V*_9_(*x*, *y*, 9.35*f*) with a factor of *k* = 9 shows moderate-quality scenes overall.

Consequently, adjusting the magnification factor *k* as an independent parameter in the proposed method enables to reconstruct a group of volumes from an elemental image array, whereas the conventional method reconstructs a volume since the magnification factors depend on their reconstruction positions. This computer simulation indicates each volume has different characteristics for varying *k*. Therefore, controlling the magnification factor as an independent parameter from the reconstruction position provides a 4-D image structure that has the *x*-axis, *y*-axis, *z*-axis, and the additional *k*-axis. Additionally, the proposed method can enhance a 3-D-reconstructed image from an elemental image array by observing the 4-D image structure.

To quantitatively compare the proposed method with the previous method, two objective measures such as the peak signal-to-noise ratio (PSNR) and the structural similarity index measure (SSIM) are calculated [30]. The reconstructed images obtained from the proposed and previous method are set so that the size of the reconstructed images is the same as that of the original image [11], which is required to calculate PSNR and SSIM between an original image and the reconstructed image.

The PSNR between an original image *O*(*x*, *y*) with a size of *p* × *q* pixels and its reconstructed image *R*(*x*, *y*) is defined as

(3) $$\mathrm{PSNR}=10log_{10}\frac{255^{2}}{MSE}.$$

where

(4) $$MSE=\frac{1}{pq}\overset{p}{\underset{x=1}{\sum }}\sum_{y=1}^{q}{\left[O\left(x,y\right)-R\left(x,y\right)\right]}^{2}$$

The SSIM index is based on similarities of local luminance, contrast, and structure between an original test image and its reconstructed image [30]. The SSIM is given by

(5) $$\mathrm{SSIM}\left(x,y\right)=\frac{\left(2\mu_{x}\mu_{y}+C_{1}\right)\left(2\sigma_{xy}+C_{2}\right)}{\left(\mu_{x}^{2}+\mu_{y}^{2}+C_{1}\right)\left(\sigma_{x}^{2}+\sigma_{y}^{2}+C_{2}\right)},$$

where *μ_x_*, *σ_x_*, and *σ_xy_* are the mean, standard deviation, and cross-covariance between *x* and *y*, respectively. The constants *C*_1_ and *C*_2_ are used to avoid instability when the means and variances become small.

Table 1 and Table 2 provide the PSNR and SSIM results from the simulations above. For most depth, the PSNR results with different *k* obtained from the proposed method show that PSNR increases as the factor *k* decreases. Additionally, PSNR approaches the PSNR from the previous method, as *k* increases. For example, for a depth of *z* = 9.35*f*, the reconstructed images for the object ‘car’ with a factor of *k* = 1 and 2 from the proposed method show 34.57 and 33.73 dB, which is 1.73 and 0.94 dB improvements, compared to the previous method. Similarly, the improved SSIMs are 0.66 and 0.55 for the same case, which is 0.44 and 0.33 improvements.

These results are because reconstructed images can suffer from unwanted signals from occluding objects in overlapping. This situation is also seen in the case of the SSIM index, as shown in Table 2, such that the smaller *k*, the higher SSIMs for the reconstructed images with the proposed method. Therefore, those results indicate that the visual qualities of the reconstructed images are improved by controlling the factor of *k* in the proposed method.

## 5. Optical Experiments

**Figure 13:**
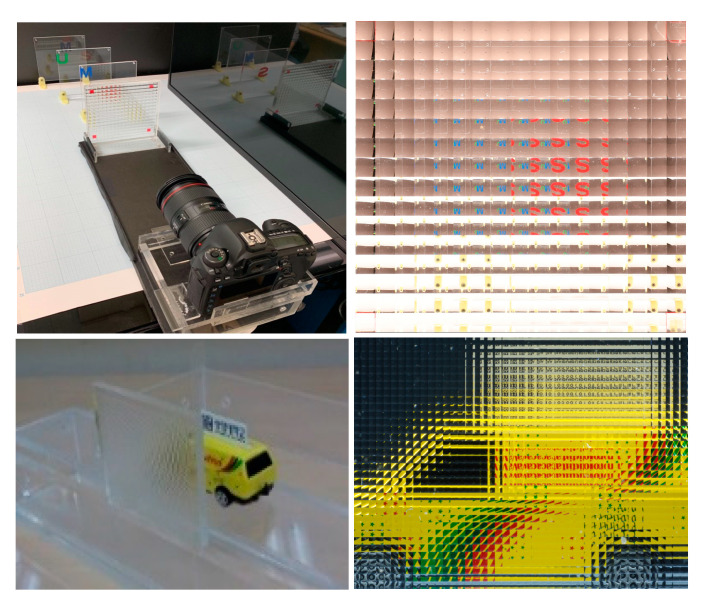
Experimental setup and captured EIAs.

To show the possibility of practical implementation of our method, we also carried out optical experiments with real 3-D objects of ‘S’, ‘M’, ‘U’, and ‘car’ to match our computer simulations in the previous section. The optical experimental setup and the elemental image arrays of the real objects are shown in Figure 13, where the optical pickup includes two lens arrays. The lens pitch of the lens array for ‘S’, ‘M’, and ‘U’ is 7.47 mm and the focal length of each lens is *f* = 73 mm. Its captured EIA has 17 × 16 elemental images, and the resolution of each elemental image is 200 × 200 pixels. The locations of the test objects away from the lens array are 200, 450, and 620 mm. The lens pitch of the lens array for ‘car’ is 1.08 mm and its focal length is 5.2 mm. The EIA for ‘car’ has 45 × 34 elemental images, and each elemental image consists of 58 × 58 pixels. The location of the ‘car’ away from the lens array is around 20 mm.

**Figure 14:**
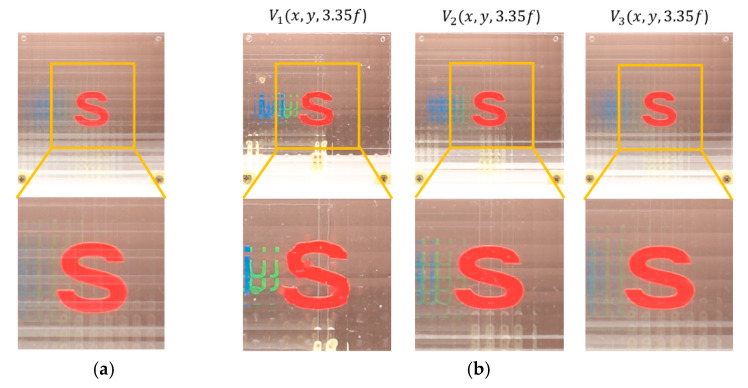
Reconstructed images for the object ‘S’ at a depth of *z* = 3.35*f* with (**a**) conventional CIIR [11], (**b**) proposed CIIR.

**Figure 15:**
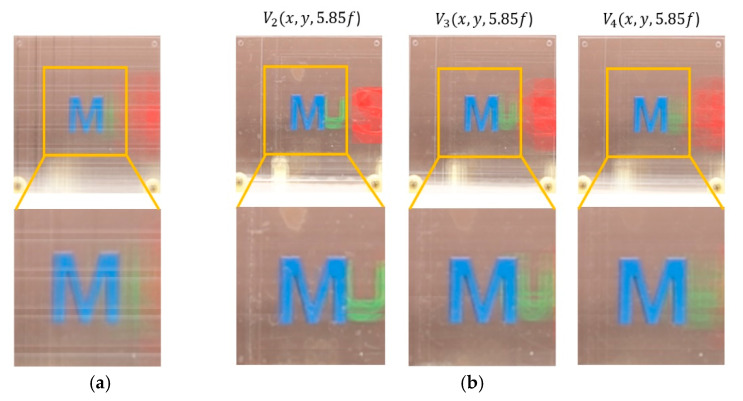
Reconstructed images for the object ‘M’ at a depth of *z* = 5.85*f* with (**a**) conventional CIIR [11], (**b**) proposed CIIR.

**Figure 16:**
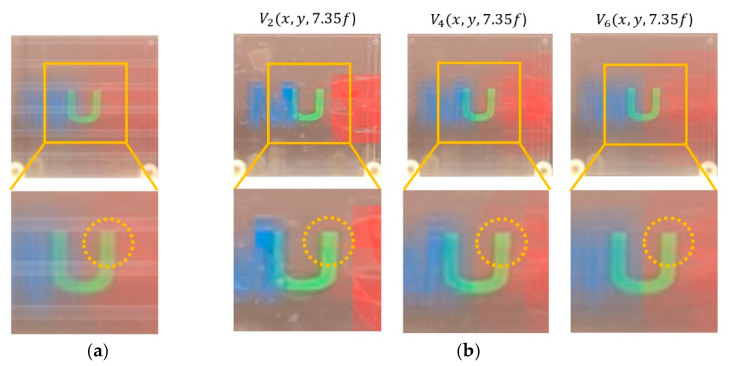
Reconstructed images for the object ‘U’ at a depth of *z* = 7.35*f* with (**a**) conventional CIIR [11], (**b**) proposed CIIR.

**Figure 17:**
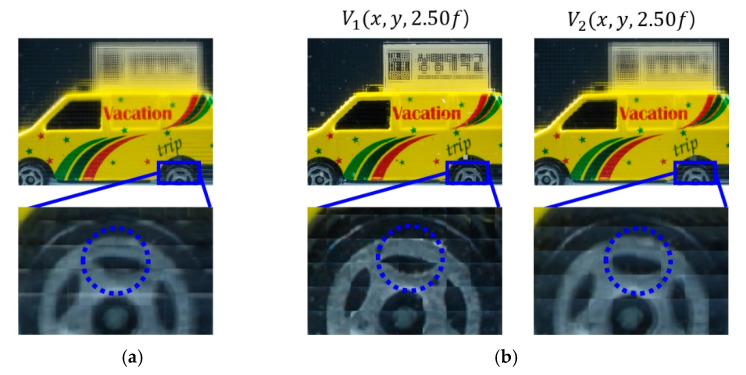
Reconstructed images for the object ‘car’ at a depth of *z* = 2.50*f* with (**a**) conventional CIIR [11], (**b**) proposed CIIR.

**Figure 18:**
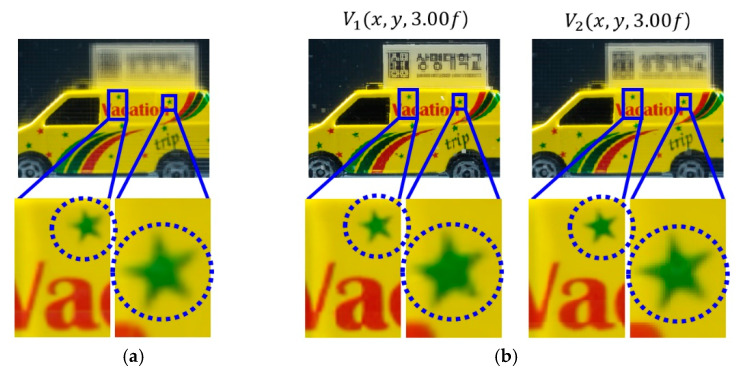
Reconstructed images for the object ‘car’ at a depth of *z* = 3.00*f* with (**a**) conventional CIIR [11], (**b**) proposed CIIR.

**Figure 19:**
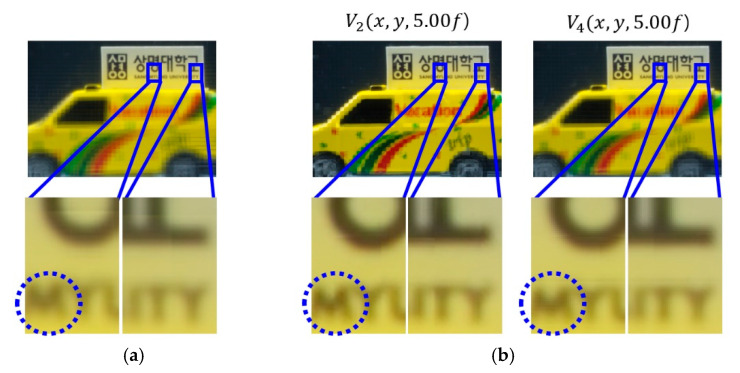
Reconstructed images for the object ‘car’ at a depth of *z* = 5.00*f* with (**a**) conventional CIIR [11], (**b**) proposed CIIR.

A various set of reconstructed images from optical experiments is provided to visually compare the proposed method with the conventional method [11], as shown in Figure 14, Figure 15, Figure 16, Figure 17, Figure 18 and Figure 19. In optical experiments, the elemental image arrays from a lens array normally suffer from noises, aberrations, and lens distortions due to the optical limitations of the lenses. Thus, the quality of the reconstructed images decreases compared with that of the computer simulation. Especially, the reconstructed images of the conventional method have a grid-shaped artifact, which is possibly caused by lens aberrations or the barrel distortion of the lens array, as illustrated in Figure 14a, Figure 15 and Figure 16a. On the contrary, our method can remove artifacts in elemental images by cropping because the distortions are more severe in the boundary than the center of each lens.

The reconstructed images according to *k* are depicted in Figure 14, Figure 15, Figure 16, Figure 17, Figure 18 and Figure 19b, compared with those from the previous method in Figure 14, Figure 15, Figure 16, Figure 17, Figure 18 and Figure 19a. The results from Figure 14, Figure 15, Figure 16, Figure 17, Figure 18 and Figure 19b indicate also that controlling the overlapping number *k* works in controlling visual quality in the real optical environment. For example, the reconstructed images highlighted with circles in Figure 16, Figure 17, Figure 18 and Figure 19 indicate that our computational reconstruction method is more robust to optical limitations than the conventional method, as well as the visual quality of reconstructed images is improved by controlling the factor of *k* in the proposed method. For another example, as shown in the middle of Figure 16, Figure 17, Figure 18 and Figure 19, the reconstructed images of our method with a lower factor of *k* have a wider depth of field than those of the previous method. Therefore, the proposed method enhances the image quality of 3-D-reconstructed images by providing a 4-D image structure with the adjustable magnification factor from a single elemental image array.

## 6. Conclusions

This paper has described a computational reconstruction method that enables to produce a 4-D image structure by introducing a free parameter *k* to control overlapping in back projection. A transformation between EIAs consisting of cropping and translating elemental images has been proposed to introduce the new parameter. Our analysis has said that the adjustable parameter is implemented from the cropping ratio that is independent of the depth. Based on this, the proposed method reconstructed a group of 3-D images or a 4-D image structure from a single elemental image array. The results from our computer simulations and optical experiments indicated the adjustable parameter works well to control the visual quality and the reconstructed images from the proposed method are superior to the conventional method in terms of image quality and visual information. Therefore, we expect the 4-D image structure from our method can be applied to acquiring more visual information from a single EIA as well as improving reconstructed images in integral imaging. Besides, we expect more studies on applying the 4-D image structure to other 3-D imaging applications.

## Figures and Tables

**Table 1:** PSNR results of conventional and proposed methods.

PSNR			‘S’, ‘M’, ‘U’			‘Leaves’, ‘Car’		
			*z*					
			3.70*f*	7.24*f*	9.35*f*	3.70*f*	7.24*f*	9.35*f*
**conventional method**			**56.77**	44.84	47.86	41.94	39.37	32.79
**proposed method**	***k***	1	57.25	**48.40**	**51.06**	42.10	39.40	**34.57**
		2	59.69	47.53	49.82	**42.21**	39.44	33.73
		3	**60.44**	46.19	49.94	42.13	39.66	33.29
		4	n/a	46.03	49.07	n/a	39.83	33.11
		5	n/a	45.97	48.80	n/a	**39.98**	32.99
		6	n/a	45.60	48.72	n/a	39.98	32.90
		7	n/a	45.03	48.52	n/a	39.96	32.82
		8	n/a	n/a	48.45	n/a	n/a	32.77
		9	n/a	n/a	48.23	n/a	n/a	32.78

**Table 2:** SSIM results of conventional and proposed methods.

SSIM			‘S’, ‘M’, ‘U’			‘Leaves’, ‘Car’		
			*z*					
			3.70*f*	7.24*f*	9.35*f*	3.70*f*	7.24*f*	9.35*f*
**conventional method**			**0.74**	0.78	0.74	0.34	0.39	0.22
**proposed method**	***k***	1	**0.91**	**0.92**	**0.92**	**0.65**	**0.67**	**0.66**
		2	0.84	0.89	0.89	0.48	0.59	0.55
		3	0.78	0.87	0.87	0.38	0.52	0.45
		4	n/a	0.85	0.84	n/a	0.47	0.38
		5	n/a	0.83	0.83	n/a	0.44	0.33
		6	n/a	0.81	0.81	n/a	0.42	0.30
		7	n/a	0.78	0.79	n/a	0.40	0.28
		8	n/a	n/a	0.77	n/a	n/a	0.24
		9	n/a	n/a	0.74	n/a	n/a	0.22
